# Communicable to Non-communicable Disease Pandemic in the Making: An Urgent Call for Post-COVID-19 Preparedness

**DOI:** 10.7759/cureus.27453

**Published:** 2022-07-29

**Authors:** Raktim Swarnakar, Shiv L Yadav

**Affiliations:** 1 Physical Medicine and Rehabilitation, All India Institute of Medical Sciences, New Delhi, New Delhi, IND

**Keywords:** public health care, public health preparedness, communicable & non communicable diseases, covid-19, covid-19 retro

## Abstract

We have entered the third year of the coronavirus disease 2019 (COVID-19) pandemic. If we look back, we can see how this pandemic caused a wide spectrum of disabilities and death worldwide. Moreover, COVID-19 is notorious for affecting multiple systems of our body leading to what we call "long-COVID". Many people are still suffering from persistent symptoms of long-COVID. Apart from respiratory complications, it is causing cardiac issues, renal failure, stroke, etc. Due to such multiple complications, the rate of disability and functional impairments has increased in the past two years following the beginning of this pandemic. Thus, an infectious disease/communicable disease such as COVID-19 is indirectly leading to increased incidence of several non-communicable diseases (cardiac, renal, neurological, etc.). In this scenario, urgent preparedness in all aspects is warranted to control such a situation.

## Editorial

We have experienced multiple waves of this communicable disease pandemic named coronavirus disease 2019 (COVID-19). Worldwide, it has caused a huge number of cases and deaths. On the other hand, a large proportion of the infected population has recovered from this infection as well.

COVID-19 is a communicable disease caused by severe acute respiratory syndrome coronavirus-2 (SARS-CoV-2). In this communicable disease pandemic, preventive medicine and critical care medicine are playing an immense role to save lives besides basic and translational research. Basic preventive approaches like proper hand washing, sanitization, masks, and physical distancing are still of utmost importance; also, vaccines can effectively prevent severe diseases. Presently numerous vaccine and drug trials are going on across the globe in the quest to find a solution to end this pandemic. Furthermore, when we are thinking of ending this pandemic, it is important to acknowledge that the impact of the pandemic would not be so easy to erase in a short period of time.

There are a large number of people who despite having recovered from the infection are facing multiple health-related issues. This is regarded as a post-COVID-19 syndrome or "long-COVID" [1]. As long as the communicable disease pandemic continues to grow, the number of long-COVID cases invariably would rise. Long-COVID is known to impact negatively multiple organ systems in the body. Such cases of multi-organ involvement result in various cardiopulmonary, neuromuscular, haematological, and gastrointestinal sequelae [1]. Therefore, it undoubtedly needs collaborative efforts from all branches of medicine and, most importantly, from rehabilitation medicine as an essential component in care delivery. Rehabilitation has immense importance in non-communicable diseases (NCDs) and also in acute and chronic care of COVID-19. It is, thus, imperative that rehabilitation should be given timely priority if it needs.

At present, 71% of all deaths occur due to NCDs worldwide [2] and, furthermore, NCDs are considered a risk factor (co-morbidity) for severe disease in SAR-CoV-2 infection. Long-COVID is contributing to a new set of NCDs in the already existing NCDs population. Multiple mental health issues and musculoskeletal complications are on the rise during the pandemic, and many people are developing these musculoskeletal issues due to reduced physical activity. It is conspicuous that physical inactivity impacts negatively on people with NCDs irrespective of COVID-19, and this pandemic accelerated this negative impact.

With the ageing population, longer life expectancies and multiple co-morbidities populations living with health burdens or NCDs are in an increasing trend. NCDs, in fact, act as a health and economic burden at the individual, community, country, and global levels [2]. Routine care for people with already existing NCDs has been disrupted globally due to pandemics and lockdown and it is mostly persons with disability who have faced such problems. Disruption of NCDs services is mostly due to cancellation of elective care, staff deployment to COVID-19 care, closure of outpatient department, lack of availability of inpatient beds during the peak time of rising COVID-19 cases, difficulty in transport during the lockdown, local rules and regulation of government, etc. [3].

**Figure 1:**
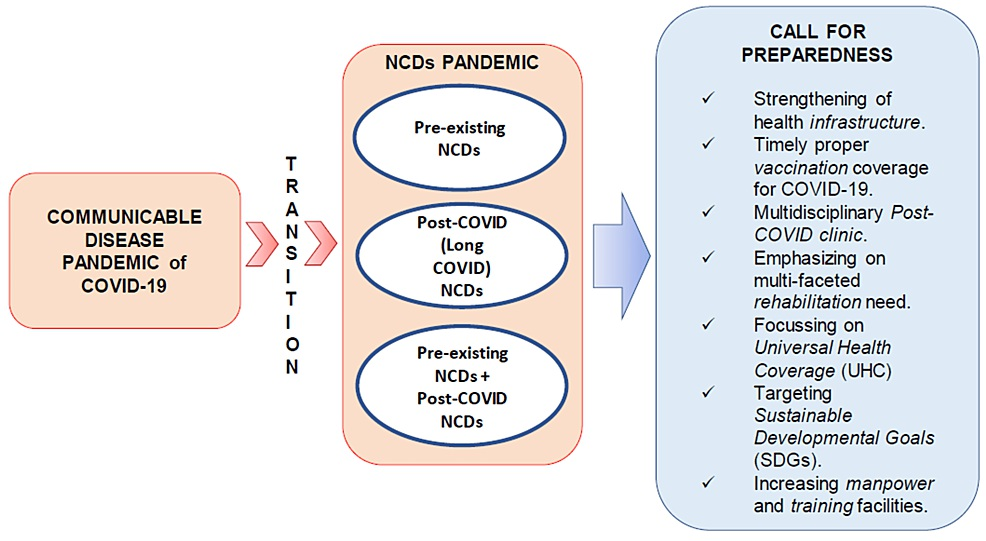
Schematic diagram showing transition between communicable disease pandemic (like COVID-19) and noncommunicable disease (NCD) pandemic and its preparedness. COVID-19: Coronavirus disease 2019; NCD: Non-communicable disease

Following the pandemic, there are three kinds of populations with NCDs as shown in Figure 1. First, those who previously had NCDs, second, those who had NDCs already and are now complicated by long-COVID sequelae following SARS-CoV-2 infection, and lastly, people who had no previous NCDs but NCDs developed after SARS-CoV-2 infection. Thus, it may contribute to an NCDs pandemic [4]. It is also interesting to note that there is a possibility to have become infected with SARS-CoV-2 a second time by different variants and in such a scenario post-COVID-19 complications following a first-time infection might possibly act as a risk factor or comorbidity for severe disease during a second infection.

We need to address this issue at the earliest, before it becomes an unbearable load on the healthcare system. Starting separate multidisciplinary long-COVID clinics or outpatient department would play an essential role to cater proper services for long-COVID NCDs. Even multidisciplinary indoor services would be needed for those who require admission. Currently, we need simultaneous planning to control communicable and non-communicable disease pandemics so that we can control the transmission and transition at the same time. We currently have no long-term scientific evidence regarding the impact of this pandemic on the healthcare system or on people with long-COVID syndrome and on those with preexisting NCDs. Since we have only passed two and a half years of the pandemic, we are still in the infancy of understanding long-COVID.

A multi-faceted preparedness in terms of strengthening the healthcare system, improving healthcare infrastructure, better government policies, increasing manpower and their training facilities, and timely full coverage of COVID-19 vaccination is one of the key unmet needs to combat such unprecedented pandemics of any kind. Focusing on Universal Health Coverage (UHC) is another important area to work on to tackle any future pandemics, especially in low-and middle-income countries [3]. Simultaneously, we should aim at the Sustainable Development Goals (SDGs), which are targeted to reduce premature mortality by one-third by 2030 (relative to 2015 levels) and plan to promote mental health [5].

Before it gets too late, we must act now to build a post-COVID-19 "fairer, healthier world".

## References

[1] Nalbandian A, Sehgal K, Gupta A (2021). Post-acute COVID-19 syndrome. Nat Med.

[2] (2022). World Health Organization: Noncommunicable diseases. https://www.who.int/health-topics/noncommunicable-diseases#tab=tab_1.

[3] Verguet S, Hailu A, Eregata GT, Memirie ST, Johansson KA, Norheim OF (2021). Toward universal health coverage in the post-COVID-19 era. Nat Med.

[4] Unwin N, Mugusi F, Aspray T (1999). Tackling the emerging pandemic of non-communicable diseases in sub-Saharan Africa: the essential NCD health intervention project. Public Health.

[5] (2022). United Nations Transforming our world: the 2030 agenda for sustainable development. Transforming Our World: The 2030 Agenda For Sustainable Development.

